# Evaluation of α-Glucosidase Inhibition and Antihyperglycemic Activity of Extracts Obtained from Leaves and Flowers of *Rumex crispus* L.

**DOI:** 10.3390/molecules28155760

**Published:** 2023-07-30

**Authors:** Dolores G. Aguila-Muñoz, Fabiola E. Jiménez-Montejo, Víctor E. López-López, Aarón Mendieta-Moctezuma, Jorge Rodríguez-Antolín, Jorge Cornejo-Garrido, María C. Cruz-López

**Affiliations:** 1 Centro de Investigación en Biotecnología Aplicada, Instituto Politécnico Nacional, Tlaxcala 90700, Mexico; fejimenezm@ipn.mx (F.E.J.-M.); vlopezyl@ipn.mx (V.E.L.-L.); amendietam@ipn.mx (A.M.-M.); 2 Centro Tlaxcala de Biología de la Conducta, Universidad Autónoma de Tlaxcala, Tlaxcala 90070, Mexico; jorge.rodrigueza@uatx.mx; 3 Laboratorio de Biología Celular y Productos Naturales, Escuela Nacional de Medicina y Homeopatía, Instituto Politécnico Nacional, Ciudad de México 07320, Mexico; jcornejog@ipn.mx

**Keywords:** *Rumex crispus* L., *α*-glucosidase inhibitors, antihyperglycemic activity, diabetes, bioassay-guided study

## Abstract

Among antihyperglycemic drugs used for treating diabetes, α-glucosidase inhibitors generate the least adverse effects. This contribution aimed to evaluate the potential antidiabetic activity of *Rumex crispus* L. by testing its in vitro α-glucosidase inhibition and in vivo antihyperglycemic effects on rats with streptozotocin (STZ)-induced diabetes. Better inhibition of α-glucosidase was found with the methanol extract versus the n-hexane and dichloromethane extracts. The methanol extract of the flowers (RCFM) was more effective than that of the leaves (RCHM), with an IC_50_ of 7.3 ± 0.17 μg/mL for RCFM and 112.0 ± 1.23 μg/mL for RCHM. A bioactive fraction (F89s) also showed good α-glucosidase inhibition (IC_50_ = 3.8 ± 0.11 μg/mL). In a preliminary study, RCHM and RCFM at 150 mg/kg and F89s at 75 mg/kg after 30 days showed a significant effect on hyperglycemia, reducing glucose levels (82.2, 80.1, and 84.1%, respectively), and improved the lipid, renal, and hepatic profiles of the rats, comparable with the effects of metformin and acarbose. According to the results, the activity of *R. crispus* L. may be mediated by a diminished rate of disaccharide hydrolysis, associated with the inhibition of α-glucosidase. Thus, *R. crispus* L. holds promise for the development of auxiliary drugs to treat diabetes mellitus.

## 1. Introduction

Diabetes, a chronic disease characterized by hyperglycemia, occurs due to insulin resistance or the lack of production of insulin. It is often associated with disorders related to the metabolism of fat, carbohydrates, and proteins [1]. Diabetic patients frequently suffer from complications caused by hyperglycemia, such as cardiovascular disorders, renal failure, neuropathy, lipid metabolism disorders, common infections, and cancer. The resulting decrease in the quality of life, in some cases leading to death [2,3], emphasizes the importance of controlling the blood glucose level of diabetic patients.

According to the International Diabetes Federation (IDF), the global prevalence of diabetes in 2019 was estimated at 9.3% of the world population (463 million people). It is projected to rise to 10.2% (578 million individuals) by 2030 and 10.9% (700 million subjects) by 2045 [4].

Drugs for controlling hyperglycemia have generally been associated with adverse effects, including the inhibition of liver regeneration, weight gain, osteoporosis, and increased cardiovascular episodes [5]. However, one therapeutic mechanism for the treatment of diabetes is considered to have null adverse effects because its activity is not involved in modulating complex biochemical processes but instead limited to the gut [6,7,8,9,10]. This mechanism, the inhibition of α-glucosidase, triggers a reduction in the digestion and absorption of dietary carbohydrates and in turn in the postprandial level of blood glucose and the demand for insulin. Especially interesting is the development of phytochemicals, mainly phenolic compounds capable of inhibiting α-amylase and α-glucosidase enzymes, as new antidiabetic agents [11,12,13].

*Rumex* L. (Polygonaceae) plants have been widely used to treat various diseases and ailments, including cancer and diabetes, due to their diversity of biologically active compounds. The roots, leaves, and fruit of *Rumex crispus* L. have been used in traditional medicine as a tonic, laxative, antipyretic, expectorant, antitussive, and vermicide, as well as for treating skin disorders, jaundice, gastrointestinal diseases, venereal diseases, diabetes, gonorrhea, and eye infections. In addition, the young leaves are eaten in soups and salads, and the seeds are used in baking cakes and as a coffee substitute [14,15,16,17]. Curiously, almost no information is available on the therapeutic potential of this plant as an antidiabetic agent. Hence, this study aimed to evaluate the effect of the leaves and flowers of *R. crispus* L. as an in vitro inhibitor of ⍺-glucosidase and an in vivo antihyperglycemic agent in rats with streptozotocin (STZ)-induced diabetes.

## 2. Results

### 2.1. Plant Material and Preparation of the Extracts

The yields (*w*/*w*) of the extraction were affected by both the plant organ being processed and the polarity of the solvent (Table 1). They were 0.75 to 0.95% for the extracts obtained with n-hexane (low polarity), 0.75 to 1.11% with dichloromethane (DCM; medium polarity), and 4.84 to 11.19% with methanol (high polarity). The methanol extract of the flowers (RCFM) afforded the highest yield, followed by the methanol extract of the leaves (RCHM).

### 2.2. Quantitative Phytochemical Analysis

The total phenolics, polyphenols, flavonoids, condensed tannins, sterols, saponins, and alkaloids of extracts of the leaves and flowers of *R. crispus* L. were quantified and are presented in Table 2. The content of total phenolics and polyphenols was considerably higher in RCHM and RCFM (260.76 ± 1.77 and 662.31 ± 4.51 mg EGA/g, and 108.65 ± 0.46 and 837.44 ± 7.50 mg ETA/g, respectively), while RCHD and RCFD recorded the highest concentration of flavonoids (190.57 ± 0.74 and 135.41 ± 0.60 mg EQ/g, respectively) and total sterols (329.82 ± 5.19 and 290.54 ± 1.15 mg EChol/g, respectively). The highest content of condensed tannins was recorded for RCHD and RCFM (122.02 ± 0.59 and 117.30 ± 0.59 mg EC/g, respectively). In addition, RCFD and RCFM exhibited the highest concentration of total saponins (374.44 ± 1.54 and 727.72 ± 2.72 mg ED/g, respectively). Quantitative estimation of the alkaloid content indicated that RCHH and RCFH had higher concentrations (2.13 ± 0.23 and 1.15 ± 0.28 mg EA/g, respectively).

In separating RCHM and RCFM from *R. crispus* L., organic solvents of ascending polarity were used to obtain fractions with less chemical complexity, increase the concentration of certain compounds, and improve the inhibitory effect. The inhibitory activity of the fractions and subfractions is detailed in Table 3.

### 2.3. α-Glucosidase Inhibition Provided by R. crispus L. and Its Fractions

RCHM and RCFM exhibited the best inhibitory activity of the extracts (Table 3), with IC_50_ values of 112.0 ± 1.23 and 7.3 ± 0.17 µg/mL, respectively. As can be appreciated, the solvent with high polarity (methanol) afforded extracts having more desirable properties than the solvents with low and medium polarity (n-hexane and DCM, respectively). Furthermore, RCFM exerted a greater inhibitory effect than RCHM, and both were more potent against α-glucosidase than acarbose (3698.0 ± 76.5 µg/mL).

Since the IC_50_ values of RCHM-FM and RCFM-FM (98.2 ± 0.78 and 4.4 ± 0.03 μg/mL) were lower than those of the crude extracts, their bioactive compounds are likely to have medium-to-high polarity. The IC_50_ values of RCFM-FM (3.89 ± 0.06 μg/mL) and F89s (3.8 ± 0.11 μg/mL) were similar, suggesting that these enriched fractions maintain the biological effect.

A Pearson correlation analysis was performed to investigate the relationship between the content of phenolic compounds and the α-glucosidase inhibitory activity of *R. crispus* L. The extracts showed a strong positive correlation between the phenolic and polyphenol content and the enzymatic activity, with an R value of 0.946 and 0.997, respectively (*p* < 0.05) (Table 4). These findings suggested that phenolic compounds may be involved and play an important role in the α-glucosidase inhibitory activity of the plant.

### 2.4. Preliminary Identification of Compounds in the Active Extracts of R. crispus L. by UHPLC-MS/MS

RCHM, RCFM, and F89s exhibited the most significant α-glucosidase inhibitory activity as compared to the other extracts. Hence, *R. crispus* L. methanolic extracts and its bioactive fraction were subjected to UHPLC-MS/MS analysis to identify the metabolites present in order to obtain better insight into the chemical constituents that could be contributing to the activity.

Several compounds from the classes of phenolic acid (gallic acid), flavan-3-ols (catechin, epicatechin), flavones (vitexin), flavonols (rutin, quercitrin, kaempferol), and anthraquinones (emodin) have been previously reported to be present in *R. crispus* L. [15,18,19]. In this study, 27 compounds were putatively identified (Table 5) based on their molecular ions in the full-scan mass spectra, retention time, fragmentation patterns, and mass error supported with databases, such as ChemSpider databases (http://www.chemspider.com/, accessed on 1 June 2023), the Natural Products Atlas (https://www.npatlas.org/, accessed on 1 June 2023), and the Waters Screening Library (UNIFI 1.8) (Waters Corporation, Milford, MA, USA). The base peak chromatogram is shown in Supplementary Material (Figures S1–S3).

Among the main metabolites of *R. crispus* L., we identified flavan-3-ols, flavonols, flavones, isoflavones, and proanthocyanidins. In the bioactive fraction F89s and in RCFM, the peaks with the highest relative abundance (%AR) corresponded to (-)-epicatechin-3-O-gallate (10.21 and 11.22%, respectively) and quercitrin (9.12 and 9.41%, respectively). In contrast, the extract with the least inhibitory activity (RCHM) registered quercitrin (21.06%) and quercetin-7-glucuronide (8.77% AR) as the main peaks, but (-)-epicatechin-3-O-gallate represented only 0.33% AR. Therefore, the abundance of these metabolites plays a key role in the α-glucosidase inhibitory activity, and it is suggested that these active compounds could be considered as standards that allow the quantification of phytochemical markers for the development of a phytopreparation of *R. crispus* L.

### 2.5. Effect of R. crispus L. on the Blood Glucose Level and Body Weight of Rats

**Figure 1:**
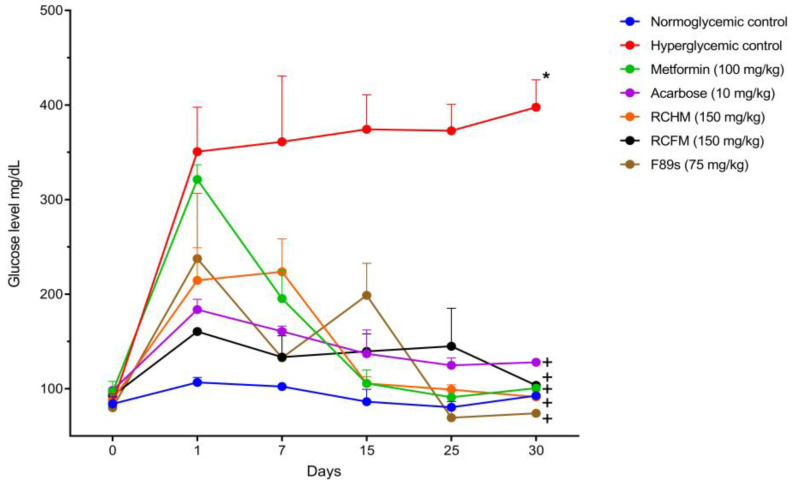
Peripheral glucose levels of normoglycemic rats and those with STZ-induced hyperglycemia and different treatments. *p* < 0.05 was considered as significant. * Statistically significant difference compared to normoglycemic controls. ^+^ Statistically significant difference compared to hyperglycemic controls.

The antidiabetic potential of *R. crispus* L. was assessed in an in vivo rat model of STZ-induced diabetes. In the groups of hyperglycemic rats treated with RCHM, RCFM, and F89s, the peripheral glucose level underwent a 57.46, 35.48, and 68.86% decrease, respectively, from the first day to the end of the 30-day treatment (Figure 1). Compared to the normoglycemic control, the glucose levels of the groups treated with *R. crispus* L. were not statistically different.

**Figure 2:**
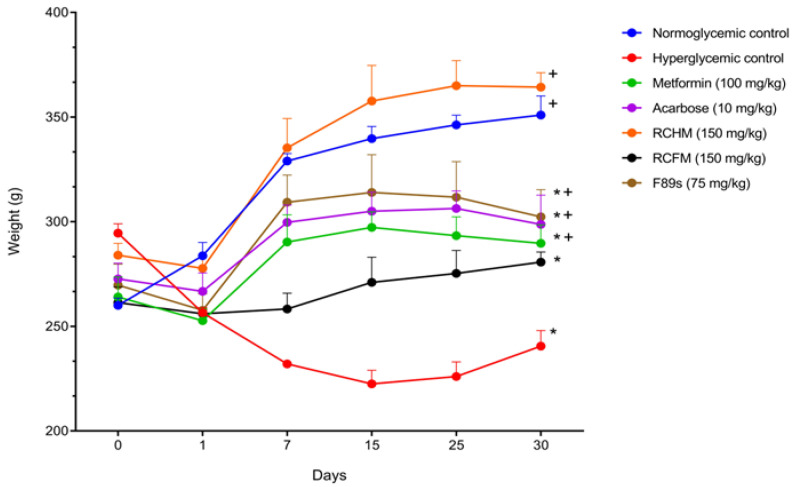
Body weight of normoglycemic rats and those with STZ-induced hyperglycemia and different treatments. *p* < 0.05 was considered as significant. * Statistically significant difference compared to normoglycemic controls. ^+^ Statistically significant difference compared to hyperglycemic controls.

The changes in the body weight of the rats were recorded during the 30-day experiment (Figure 2). During the first day post-induction of diabetes, the affected animals showed a 6.2% weight loss, while the normoglycemic controls concomitantly underwent a 23.83% weight gain. However, body weight loss was controlled to the extent that hyperglycemia was diminished after treatment with *R. crispus* L. By the end of the treatment, there was a 31.27, 9.633, and 15.73% body weight gain in rats treated with RCHM, RCFM, and F89s, respectively, while groups treated with metformin and acarbose showed a body weight gain of 14.70 and 11.9%, respectively.

### 2.6. Effect of R. crispus L. on the Serum Glucose and Insulin Levels

**Figure 3:**
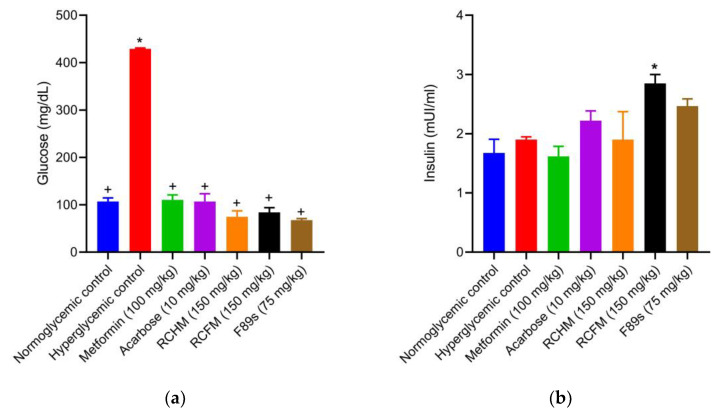
The serum levels of glucose (**a**) and insulin (**b**) of normoglycemic rats and those with STZ-induced hyperglycemia and different treatments. *p* < 0.05 was considered as significant. * Statistically significant difference compared to normoglycemic controls. ^+^ Statistically significant difference compared to hyperglycemic controls.

The serum glucose levels of rats treated for 30 days with RCHM, RCFM, and F89s decreased by 82.2, 80.1, and 84.1%, respectively, compared to the hyperglycemic controls (Figure 3a). In addition, no statistical difference existed between these groups and the normoglycemic, metformin, and acarbose groups. Compared to the hyperglycemic controls, there was a 50 and 29.8% increase in the insulin level of the groups treated with RCFM and F89s, respectively (Figure 3b). The latter two groups did not differ significantly from the normoglycemic or the acarbose group. The insulin level was 76.1% higher in the RCFM-treated animals than in the metformin group.

### 2.7. Effect of R. crispus L. on the Serum Lipid Profile

Hyperglycemic control rats showed a significant increase in the levels of TG, LDL, and VLDL and a significant decrease in TC and HDL levels compared to the corresponding values of the normoglycemic controls (Table 6). Compared to the hyperglycemic controls, the administration of RCHM, RCFM, and F89s reduced the levels of TC (by 18.9, 24.8, and 12.1%, respectively), TG (by 82.21, 82.29, and 82.38%, respectively), and VLDL (by 82.21, 82.29, and 82.38%, respectively). The concentration of HDL was significantly higher in the RCHM- and F89-treated groups (18.91 and 48.05%, respectively) but 15.88% lower in the RCFM-treated animals. LDL declined by 21.3% in the F89s-treated rats and increased by 5 and 1.2% in the RCHM and RCFM groups, respectively.

After inducing hyperglycemia with STZ, the atherogenic index (AI) of the affected rats increased significantly. Treatment with RCHM, RCFM, and F89s caused a 34.5, 14.3, and 42.1% reduction, respectively, in the AI (Table 5). The levels of AI did not differ significantly between the latter three groups and the metformin or the acarbose group.

### 2.8. Effect of R. crispus L. on the Hepatic Profile

**Figure 4:**
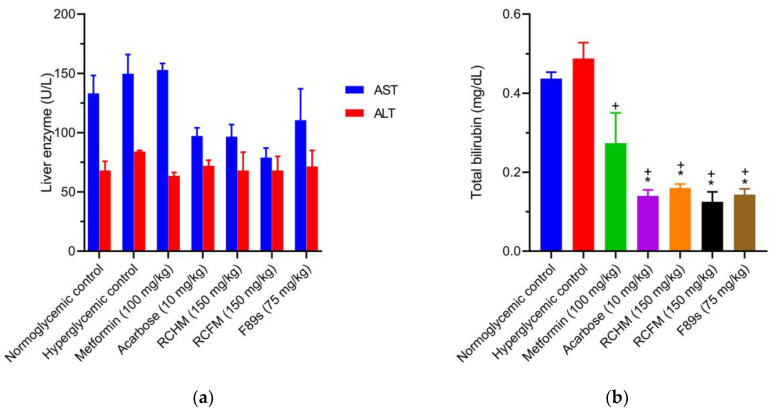
The levels of liver enzymes (**a**) and total bilirubin (**b**) in normoglycemic rats and those with STZ-induced hyperglycemia and different treatments. *p* < 0.05 was considered as significant. * Statistically significant difference compared to normoglycemic controls. ^+^ Statistically significant difference compared to hyperglycemic controls.

The levels of AST and ALT liver enzymes were elevated in the hyperglycemic controls compared to the normoglycemic controls. Taking the hyperglycemic controls as a reference, the administration of RCHM, RCFM, and F89s reduced the levels of AST (35.1, 47.2, and 26.2%, respectively) and ALT (16.5, 14.5, and 17%, respectively) (Figure 4a). In the metformin group, there was a 14.9% increment in the concentration of AST compared to the normoglycemic controls.

The levels of total bilirubin in the normoglycemic and hyperglycemic controls were 0.44 ± 0.02 and 0.49 ± 0.04 mg/dL, respectively. Compared to the hyperglycemic controls, treatment with RCHM, RCFM, and F89s decreased the concentration of bilirubin by 67.2, 74.4, and 70.6%, respectively (Figure 4b). The level of total bilirubin was not significantly different between the latter three groups and the metformin or the acarbose group.

### 2.9. Effect of R. crispus L. on the Renal Profile

**Figure 5:**
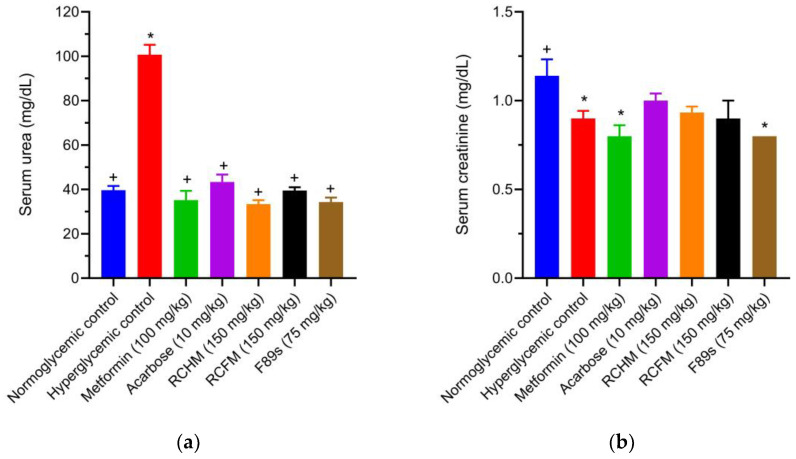
The serum levels of urea (**a**) and creatinine (**b**) in normoglycemic rats and those with STZ-induced hyperglycemia and different treatments. *p* < 0.05 was considered as significant. * Statistically significant difference compared to normoglycemic controls. ^+^ Statistically significant difference compared to hyperglycemic controls.

A higher serum urea level was found in the hyperglycemic versus normoglycemic control animals. Compared to the hyperglycemic controls, treatment with RCHM, RCFM, and F89s reduced this parameter by 66.9, 60.8, and 65.9%, respectively (Figure 5a). There was no notable difference in the urea level of the metformin-, acarbose-, and *R. crispus* L.-treated animals. Regarding the serum creatinine level, no significant differences existed between the normoglycemic and hyperglycemic controls (Figure 5b).

## 3. Discussion

The methanol extracts of the leaves and flowers of *R. crispus* L. were found to inhibit the α-glucosidase enzyme. In a preliminary study, these same extracts showed similar effects as metformin and acarbose in a rat model of STZ-induced hyperglycemia. The plant organ being processed and the solvent influenced the effect of the extracts, presumably by determining the secondary metabolites present.

Regarding the solvent, its polarity affects the extraction of chemical constituents and thus produces distinct bioactivity. The methanolic extracts provided a good inhibitory effect on α-glucosidase, while low inhibitory activity was displayed by the n-hexane and DCM extracts. In contrast, the yields were higher for the methanolic extracts of flowers (RCFM) than of leaves (RCHM), the latter of which had a lower IC_50_ value than that of acarbose. Although RCFM was the most active extract, its effect was not significantly different from that of RCFM-FM, RCFM-SA, and F89s. The fractionation may have favored the enrichment of the bioactive compounds in the fractions.

Considering the extraction method, it is inferred that the main chemical constituents of *R. crispus* L. are phenolic compounds, in agreement with previous studies reporting variation in the content of phenolic compounds in plant organs, in the following order (in both flowering and fruiting stages): generative part (flowers, seeds) > leaves > root > stem [31,32]. In addition, the quantitative analysis showed that *R. crispus* L. leaves and flowers had variable amounts of phytochemicals in the methanolic, dichloromethane, and hexane extracts. However, the highest content of total phenols and polyphenols was obtained in the methanolic extracts. Likewise, the extracts of medium polarity (RCHD and RCFD) presented a higher concentration of flavonoids and condensed tannins, which is in accordance with Idris et al. [33], who reported that the total methanolic extract of the leaves of *R. crispus* L. exhibits the highest content of phenolic compounds (103.54 ± 3.70 mg Eq gallic acid/g) and the acetone extract exhibits the highest content of flavonoids (526.23 ± 17.52 mg Eq quercetin/g), flavonols (558.25 ± 12.53 mg Eq quercetin/gE), and proanthocyanidins (300.77 ± 14.21 mg Eq catechin/gE). These results indicate that *R. crispus* L. is an important source of phenolic compounds.

The extracts showed a high correlation between the phenolic compounds and enzymatic activity, suggesting that the significant α-glucosidase inhibitory activity in polar extracts is attributable to the presence of phenolic compounds and flavonoids and their glycosides, such as (-)-epicatechin-3-O-gallate, quercitrin, and quercetin-7-glucuronide. Previously, the capacity of phenolic compounds, such as flavonoids from *Morus atropurpurea*, *Scutellaria baicalensis*, *Morinda lucida*, *Momordica charantia*, and *Moringa oleifera*, to act as α-glucosidase inhibitors has been documented in the literature [34,35,36,37].

Regarding the antidiabetic potential of the genus *Rumex*, the methanolic extracts from the leaves and flowers of *R. maderensis* and from the root of *R. crispus* L. are reported to inhibit carbohydrate- and lipid-metabolizing enzymes [38,39]. Phenolic compounds from *R. scutatus*, *R. dentatus,* and *R. patientia* lowered hyperglycemia and improved the glucose tolerance, insulin sensitivity, and even the lipid profile and the hepatoprotective effect in diabetic rats [40,41,42]. These improvements were presently observed after the administration of RCHM, RCFM, and F89s. Therefore, the antihyperglycemic action of *R. crispus* L. can be attributed to greater glucose absorption stemming from a higher level of insulin. Additionally, a better lipid profile resulted from a decrease in the levels of TC, TG, LDL, and VLDL and an increase in the level of HDL. Likewise, liver and kidney markers indicated a protective effect of *R. crispus* L.

## 4. Materials and Methods

### 4.1. Plant Material and Preparation of the Extracts

The leaves and flowers of the plant were collected in the State of Tlaxcala, Mexico (in 2017 and 2019, 19,232,715 and 98,257,153). Authentication was carried out by the Herbarium of the Benemérita Universidad Autónoma de Puebla (HBUAP, State of Puebla, Mexico, voucher number HUAP 0072233). The dried and pulverized plant material was extracted with consecutive maceration using n-hexane, DCM, and methanol in a 1:3 weight/volume ratio. The solvent was changed every 24 h for three days at room temperature. The resulting extracts were filtered and concentrated in a rotary evaporator at reduced pressure, then lyophilized, and stored at −4 °C.

### 4.2. Quantitative Phytochemical Analysis

The total phenol content was evaluated using the Folin–Ciocalteu method adapted to a 96-well microplate [43]. Briefly, 75 μL of the extract was added to 750 μL of solution A (50 mL of 2% Na_2_CO_3_, 1 mL of 1% CuSO_4_, and 1 mL of 2.7% KNaC_4_H_4_O_6_) and 150 μL of 0.5 N NaOH. After 10 min incubation at room temperature and 200 rpm, 75 μL of 1 N Folin–Ciocalteu reagent was added. The reaction mixture was incubated for 30 min at room temperature. Subsequently, absorbance was recorded at 750 nm in a microplate reader (Epoch BioTek^®^ Instruments, Winooski, VE, USA). Gallic acid was used as a standard, and the total phenolic content was determined and reported as micrograms equivalent to gallic acid per gram of extract (mg EGA/g).

The total polyphenol content was determined using the Folin–Ciocalteu method adapted to a 96-well microplate [44]. The reaction mixture containing 400 μL of 1 M Na_2_CO_3_, 100 μL of the extract, and 500 μL of 2 N Folin–Ciocalteu reagent (1:9) was incubated for 20 min at room temperature. Afterward, absorbance was recorded at 765 nm in a microplate reader (Epoch BioTek^®^). Tannic acid was maintained as a standard, and the result was expressed as micrograms equivalent to tannic acid per gram of extract (mg ETA/g).

The total flavonoid content was determined using the colorimetric method with AlCl_3_ [45] adapted to a 96-well microplate. The reaction mixture consisted of 300 μL of 98% ethanol, 20 μL of 10% AlCl_3_, 20 μL of 1 M CH_3_COOK, 560 μL of distilled water, and 100 μL of the extract. The mixture was incubated for 30 min at room temperature, and then absorbance was measured at 415 nm in a microplate reader (Epoch BioTek^®^). Quercetin was used as a standard, and the total flavonoid content was expressed as micrograms equivalent to quercetin per gram of extract (mg EQ/g).

The condensed tannin content was estimated based on the method of vanillin/HCl [46]. The reaction mixture consisted of 2 mL of a solution (1:1) of 4% vanillin/8% HCl (methanol solution) added to 400 μL of the extract. The mixture was incubated in a water bath at 30 °C for 20 min, and afterward, absorbance was measured at 550 nm in a microplate reader (Epoch BioTek^®^). Catechin was used as a standard, and the condensed tannin content was expressed as micrograms equivalent to catechin per gram of extract (mg EC/g).

The total saponin content was evaluated following the standard procedure [47]. The reaction mixture with 0.25 mL of the extract, 0.25 mL of 8% vanillin, and 2.5 mL of 72% H_2_SO_4_ was placed in a 60 °C water bath for 10 min, allowed to cool, and then transferred to an ice bath for 4 min; finally, absorbance was measured at 544 nm in a UV spectrophotometer (Genesys 10S UV-Vis). Diosgenin was used as a standard, and the results were expressed as micrograms equivalent to diosgenin per gram of extract (mg ED/g).

The total sterol content was evaluated with the Liebermann–Bürchard method [48]. The reaction mixture with 100 μL of the extract and 1 mL of Liebermann–Bürchard reagent (10 mL of concentrated H_2_SO_4_, 60 mL of acetic anhydride in an ice bath, 30 mL of acetic acid, and 0.6 g of sodium sulfate anhydrous) was placed in a water bath at 35 °C for 10 min, and then absorbance was measured at 550 nm in a UV spectrophotometer (Genesys 10S UV-Vis). For the quantification of total sterols, cholesterol was used as a standard, and the results were expressed as micrograms equivalent to cholesterol per gram of extract (mg EChol/g).

The total alkaloid content was determined using the method based on the reaction of an alkaloid with bromocresol green (BCG), as described previously [49]. The extract was dissolved in 2N HCl and then filtered. Next, 1 mL of this solution was transferred to a separatory funnel and washed with 10 mL of chloroform (3 times). The pH of this solution was adjusted to neutral with 0.1 N NaOH. Subsequently, 5 mL of BCG solution and 5 mL of phosphate buffer were added to this solution. The mixture was shaken and complexed extracted with 1, 2, 3, and 4 mL of chloroform with vigorous stirring, and then the extract was collected in a 10 mL volumetric flask and diluted with chloroform. The absorbance of the complex in chloroform was measured at 470 nm in a UV spectrophotometer (Genesys 10S UV-Vis). For the quantification of total alkaloids, atropine was used as a standard, and the results were expressed as micrograms equivalent to atropine per gram of extract (mg EA/g).

### 4.3. Preparation of R. crispus L. Fractions

Fractionation was achieved using solid–liquid extraction with solvents of different polarities (DCM, acetone, and methanol). The distinct methanolic crude extracts were suspended in DCM, shaken to dissolve them completely in the solvent, and washed with methanol. The filtrate of the fractions was concentrated in a rotary evaporator at reduced pressure to obtain the DCM and methanol fractions, which were subsequently lyophilized for proper storage. The methanolic fractions were subjected to a second series of consecutive washings, as described for the fractionation procedure with the aforementioned solvents, to obtain fractions in acetone and methanol. The acetone fraction of the methanolic extract of flowers was separated using column chromatography packed with 70–230 silica gel (Merck, Darmstadt, Germany), with mixtures of ascending polarity of n-hexane, ethyl acetate, acetone, and methanol as the mobile phase. Based on the similarity of the thin-layer chromatography (TLC) profiles, the extract was classified into 19 subfractions. The F89s subfraction was obtained as a brown solid. The different dry fractions were transferred to separate vials and stored at −4 °C until needed.

### 4.4. α-Glucosidase Inhibitory Assay

The inhibition of α-glucosidase was tested using the chromogenic method previously reported by Salehi et al. [50], with some modifications. A mixture of 20 µL of α-glucosidase type I solution (0.5 units/mL in 0.1 M phosphate buffer (pH 6.9)) from *S. cerevisiae* (Cas 9001-42-7, batch # 0000093219, Sigma-Aldrich, St. Louis, MO, USA), 120 µL of 0.1 M phosphate buffer (pH 6.9), and 10 µL of the sample was incubated in 96-well plates at 37 °C for 15 min. After preincubation, the enzymatic reaction was started by adding 20 µL of 5 mM *p*-nitrophenyl-α-D-glucopyranoside (*p*NPG) solution in 0.1 M phosphate buffer (pH 6.9) (Cas 3767-28-0, batch # BCBW3619, Sigma-Aldrich) and incubated at 37 °C for another 15 min. The reaction was stopped by adding 80 µL of 0.2 M sodium carbonate solution, and then absorbance was recorded at 405 nm in a microplate reader (Epoch, BioTek^®^). The reaction system without any test sample was used as a control, and the system without α-glucosidase served as a blank to correct for background absorbance. The inhibitory activity of the sample on α-glucosidase was calculated with the following equation:

(1) $$\%\;Inhibition=\left(1-\frac{{Sample}_{Absorbance}}{{Control}_{Absorbance}}\right)\times 100$$

### 4.5. Preliminary Identification of Phenolic Compounds Using UHPLC-MS/MS

An ACQUITY UPLC System (Waters Corporation, Milford, MA, USA) and a Xevo G2-XS QTof MS mass spectrometer (Waters Corporation, Milford, MA, USA) equipped with an Acquity BEH C18 column (2.1 mm, 100 mm, 1.7 μm) (Waters Corporation, Milford, MA, USA) were operated to identify the active extracts’ metabolites. The column temperature was maintained at 45.0 °C. The mobile phase consisted of acetonitrile (solvent A) and water (solvent B) (containing 0.05% formic acid) and was pumped at a flow rate of 0.5 mL/min. The gradient condition to equilibrate the column was: 0–2 min, 0–5% A; 2–5 min, 5–10% A; 5–8 min, 10–15% A; 8–12 min, 15–25% A; 12–15 min, 25–33% A; 15–18 min, 33–53% A; 18–20 min, 53–95% A; and 20–22 min, 95–5% A. The mass spectrometer, equipped with an electrospray source (ESI), was operated in negative ionization mode.

The MS parameters were as follows: capillary voltage, 2.5 kV; sample cone, 40 V; source temperature, 100 °C; desolvation temperature, 550 °C; cone gas flow rate, 50 L/h; and desolvation gas (N_2_) flow rate, 1000 L/h. MS spectra were acquired by full-range acquisition covering 100–1200 m/z. Dry extracts were re-dissolved (10 mg/mL) in water, followed by sonication and filtration (0.22 µm PTFE membrane filters), and injected (2 μL) into the chromatographic equipment.

### 4.6. Experimental Animals

Male Wistar rats weighing 250 ± 20 g were housed in standard cages, kept at a controlled room temperature (22 ± 2 °C) and humidity (55 ± 5%) in a 12/12 h light/dark cycle, and provided a standard diet and water ad libitum. All procedures were developed in accordance with the Mexican official guidelines for laboratory animals (NOM-062-ZOO-1999) [51] and ratified by the Bioethics Committee of the Escuela Nacional de Medicina y Homeopatía of the Instituto Politécnico Nacional (registration CB-001-2020).

### 4.7. Induction of Hyperglycemia in Rats

Hyperglycemia was induced in overnight-fasted (12 h) rats via a single intraperitoneal (i.p.) injection of 50 mg STZ/kg body weight in 0.1 M citrate buffer solution (pH 4.4). At 72 h post-injection, taken as day 1, blood glucose was measured with a glucometer (Accu-Chek^®^ Active kit, Hoffman-La Roche Ltd., Mexico City, Mexico). Only the animals with blood glucose levels greater than 150 mg/dL were considered diabetic and included in the experiments [52].

### 4.8. Experimental Design

The rats were divided into seven groups (*n* = 6 per group): group I, the normoglycemic controls (water, 300 µL); group II, the hyperglycemic controls, being the vehicle-treated diabetic animals (DMSO:H_2_O (1:3), 300 µL); groups III and IV, diabetic animals administered metformin (100 mg/kg) and acarbose (10 mg/kg), respectively; and groups V, VI, and VII, diabetic rats administered RCHM and RCFM at 150 mg/kg and F89s at 75 mg/kg, respectively, in DMSO:H_2_O (1:3) via the intragastric route every 48 h for 30 days. Upon completion of the treatment period, the animals were fasted overnight, and then they were anesthetized with xylazine and ketamine to be sacrificed via cardiac puncture according to the corresponding Mexican official guideline (NOM-033-ZOO-1995) [53].

### 4.9. Measurement of Body Weight and the Level of Peripheral Glucose

The body weight and peripheral glucose (evaluated in blood taken from the distal tail portion) were recorded weekly for each animal during the 30-day experimental period.

### 4.10. Biochemical Profile

Blood samples were extracted via cardiac puncture. The serum obtained from the blood samples (without an anticoagulant) was processed in the automated biochemical analyzer AutoKem II KontroLab-Stat Fax to quantify the following biochemical parameters: urea, creatinine, total cholesterol (TC), triglycerides (TG), high-density lipoprotein (HDL), low-density lipoprotein (LDL), very low-density lipoprotein (VLDL), aspartate aminotransferase (AST), alanine aminotransferase (ALT), the atherogenic index (AI), total bilirubin, and insulin.

### 4.11. Statistical Analysis

Results were expressed as the mean ± SEM. Statistical differences, considered at *p* < 0.05, were examined with one-way analysis of variance (ANOVA), followed by a Bonferroni post hoc test using Prism GraphPad version 8.0. Correlations among the enzymatic activities were performed with Pearson’s correlation test using Minitab software (version 16, Minitab Inc., State College, PA, USA), where IC_50_ values were converted to 1/IC_50_ values to inverse the relationship between absorbance and activity.

## 5. Conclusions

Three extracts of *R. crispus* L., RCHM, RCFM, and F89s, served as good inhibitors of α-glucosidase, thus reducing the rate of disaccharide hydrolysis. In addition, they produced an antihyperglycemic effect in rats with STZ-induced hyperglycemia, showing comparable effects to metformin and possibly through a mechanism of action similar to that of acarbose. Hence, the potential of using *R. crispus* L. to develop auxiliary drugs for the treatment of diabetes mellitus merits further research.

## Figures and Tables

**Table 1:** Physical description and yields of the crude extracts.

ID	Physical Description of the Extract	Yield (g)	% Yield (*w*/*w*)
RCHH	Dark brown and gummy	3.694	0.92
RCHD	Dark brown and gummy	4.437	1.11
RCHM	Brown powder	19.372	4.84
RCFH	Dark brown and gummy	3.5189	0.75
RCFD	Dark brown and gummy	3.069	0.65
RCFM	Brown powder	52.8	11.19

RC, *Rumex crispus* L.; plant organ subjected to extraction (H, leaves; F, flowers); solvent (H, n-hexane; D, dichloromethane; and M, methanol).

**Table 2:** Phytochemical composition of crude extracts.

ID	Total Phenolics (mg EGA/g)	Total Polyphenols (mg ETA/g)	Flavonoids (mg EQ/g)	Condensed Tannins (mg EC/g)	Total Sterols (mg EChol/g)	Total Saponins (mg ED/g)	Alkaloids (mg EA/g)
RCHH	12.24 ± 0.66 ^E^	3.54 ± 0.27 ^C^	63.18 ± 0.39 ^C^	23.62 ± 1.38 ^D^	142.82 ± 1.0 ^D^	154.14 ± 1.72 ^F^	2.13 ± 0.23 ^A^
RCHD	54.78 ± 0.83 ^C^	7.39 ± 0.17 ^C^	190.57 ± 0.74 ^A^	122.02 ± 0.59 ^A^	329.82 ± 5.19 ^A^	161.05 ± 1.54 ^E^	0.55 ± 0.22 ^CD^
RCHM	260.76 ± 1.77 ^B^	108.65 ± 0.46 ^B^	19.37 ± 0.18 ^E^	81.19 ± 0.20 ^C^	13.68 ± 1.02 ^E^	319.93 ± 0.96 ^C^	0.76 ± 0.07 ^BC^
RCFH	7.50 ± 0.20 ^E^	3.02 ± 0.10 ^C^	26.80 ± 0.22 ^D^	8.75 ± 0.14 ^F^	163.78 ± 3.06 ^C^	289.73 ± 1.62 ^D^	1.15 ± 0.28 ^B^
RCFD	38.76 ± 0.26 ^D^	9.11 ± 0.46 ^C^	135.41 ± 0.60 ^B^	21.48 ± 0.59 ^E^	290.54 ± 1.15 ^B^	374.44 ± 1.54 ^B^	0.18 ± 0.02 ^D^
RCFM	662.31 ± 4.51 ^A^	837.44 ± 7.50 ^A^	8.34 ± 0.16 ^F^	117.30 ± 0.59 ^B^	2.09 ± 0.11 ^F^	727.72 ± 2.72 ^A^	0.18 ± 0.02 ^D^

Different letters in a column indicate significant differences (*p* < 0.05) using Tukey’s test. Values are expressed as the mean ± standard deviation (*n* = 3).

**Table 3:** Inhibitory activity of the extracts and fractions of *R. crispus* L. on α-glucosidase.

ID	IC_50_ (μg/mL)	ID	IC_50_ (μg/mL)
RCHH	>400	RCFH	>400
RCHD	>400	RCFD	>400
RCHM	112.0 ± 1.23	RCFM	7.3 ± 0.17
RCHM-FDCM	205.5 ± 2.46	RCFM-FDCM	15.8 ± 0.15
RCHM-FM	98.2 ± 0.78	RCFM-FM	4.4 ± 0.03
RCHM-SA	>100	RCFM-SA	3.89 ± 0.06
RCHM-SM	>100	RCFM-SM	>4.0
Acarbose	3698.0 ± 76.5	F89s	3.8 ± 0.11

Values are expressed as the mean (based on four replicates) ± standard deviation. Acarbose served as a positive control. Fractions were obtained with dichloromethane (RCHM-FDCM and RCFM-FDCM) or methanol (RCHM-FM and RCFM-FM), while subfractions were obtained with acetone (RCHM-SA and RCFM-SA) or methanol (RCHM-SM and RCFM-SM) and the bioactive fraction F89s.

**Table 4:** Correlation matrix with the Pearson coefficient values for the phytochemicals and α-glucosidase inhibitory activity of *R. crispus* L.

Parameters	1/IC_50_	Total Phenolics	Total Polyphenols	Flavonoids	Condensed Tannins	Total Sterols	Alkaloids
Total phenolics	0.946						
Total polyphenols	0.997	0.968					
Flavonoids	−0.459	−0.522	−0.487				
Condensed Tannins	0.541	0.641	0.562	0.146			
Total Sterols	−0.588	−0.716	−0.63	0.925	−0.178		
Alkaloids	−0.434	−0.49	−0.447	−0.204	−0.504	−0.081	
Total saponins	0.914	0.895	0.92	−0.501	0.35	−0.568	−0.62

**Table 5:** Tentative identification of metabolites present in *R. crispus* L.

Peak	RT (min)	Tentative Identification	Formula	Observed *m/z*	Main Fragments (*m/z*)	Bibliography
1	0.47	Aspalathin	C_21_H_24_O_11_	451.1211	377, 271, 211, 125	
2	0.75	Gallic acid	C_7_H_6_O_5_	169.0124	151, 125, 124, 123	[20,21,22]
3	1.94	D-(+)-catechin	C_15_H_14_O_6_	289.071	273, 257, 179, 161, 137, 125	[20,21,23]
4	2.42	Procyanidin A2	C_30_H_24_O_12_	575.1205	451, 425, 407, 289, 161, 125	
5	2.73	(−)-Epicatechin	C_15_H_14_O_6_	289.0716	273, 243, 161, 137, 125, 123	[20,21]
6	3.08	Isorhamnetin-3-O-galactoside	C_22_H_22_O_12_	477.1034	411, 372, 314, 289, 250, 193	[24]
7	4.6	(-)-Epicatechin-3-O-gallate	C_22_H_18_O_10_	441.0829	301, 271, 245, 175, 169, 151, 125	[21]
9	4.74	Quercetin-7-glucuronide	C_21_H_18_O_13_	477.0667	463, 301, 300, 288, 271, 151, 133, 123, 125	[25]
8	4.74	Demethylwedelolactone	C_15_H_8_O_7_	299.0183	271, 255, 151, 133	
10	4.79	Rutin	C_27_H_30_O_16_	609.1491	463, 301, 299, 243, 151, 133	[21,26]
11	4.84	Lyoniside	C_27_H_36_O_12_	551.2143	521, 289, 215, 187, 171, 133, 125	
12	5.01	Procyanidin B3 3′-O-gallate	C_37_H_30_O_16_	729.1468	559, 407, 303, 289, 287, 269, 169, 125	[26]
13	5.02	Brucein A	C_26_H_34_O_11_	521.2023	461, 359, 313, 289, 165, 125	
14	5.25	Nudiposide	C_27_H_36_O_12_	551.2122	521, 359, 289, 284, 165, 125	
15	5.5	Tectoridin	C_22_H_22_O_11_	461.1089	447, 357, 300, 284, 169	
16	5.84	Scutellarin	C_21_H_18_O_12_	461.0732	300, 285, 284, 271, 161, 151, 133	[27]
17	6.08	Quercitrin	C_21_H_20_O_11_	447.0931	300, 285, 243, 201, 190, 151	[28]
18	6.09	Rhein glucoside	C_21_H_18_O_11_	445.0776	271, 255, 243, 215, 161, 133, 125	
19	6.36	Isorhamnetin 3-glucoside	C_22_H_22_O_12_	477.1041	407, 372, 314, 285, 243, 161, 133	
20	6.93	Aloesin	C_19_H_22_O_9_	393.1179	300, 299, 285, 270, 255, 227	
21	7.06	Vitexin	C_21_H_20_O_10_	431.0987	299, 285, 270, 255, 227, 135	[21]
22	7.37	Chrysophanol-8-(6-O-galloyl-β-D-glucopyranoside)	C_28_H_24_O_13_	567.1156	407, 341, 270, 225, 169, 135, 125	
23	7.68	Prunin	C_21_H_22_O_10_	433.1134	323, 311, 270, 269, 231, 151, 125	[21]
24	8.96	7-Hydroxy-3-(4-hydroxyphenyl)-4-oxo-4H-chromen-8-yl-hexopyranoside	C_21_H_20_O_10_	431.0983	359, 297, 269, 131	
25	9.05	Apigenin	C_15_H_10_O_5_	269.0443	253, 230, 227, 151, 131, 117	[21,27]
26	9.21	Kaempferol	C_15_H_10_O_6_	285.0391	253, 229, 227, 151, 137, 117	[21,29]
27	14.74	Emodin	C_15_H_10_O_5_	269.0446	241, 165, 137, 133	[23,30]

**Table 6:** The serum lipid profile and atherogenic index of normoglycemic rats and those with STZ-induced hyperglycemia and different treatments.

	Normoglycemic Controls	Hyperglycemic Controls	Metformin (100 mg/kg)	Acarbose (10 mg/kg)	RCHM (150 mg/kg)	RCFM (150 mg/kg)	F89s (75 mg/kg)
TC (mg/dL)	72.20 ± 2.75	64.50 ± 2.66	61.60 ± 2.97	51.8 ± 3.44 *	52.3 ± 3.76 *	48.50 ± 5.50 *	56.70 ± 2.96 *
HDL (mg/dL)	35.60 ± 2.82 ^+^	14.86 ± 1.58 *	24.20 ± 1.46 *^,+^	25.5 ± 3.52 *^,+^	17.67 ± 1.45 *	12.50 ± 2.50 *	22.00 ± 3.51 *
LDL (mg/dL)	21.67 ± 4.39	31.13 ± 0.93	30.60 ± 4.02	30.33 ± 1.43	32.67 ± 5.9	31.50 ± 7.50	24.50 ± 0.50
TG (mg/dL)	104.80 ± 12.71 ^+^	200.50 ± 21.03 *	83.29 ± 7.89 ^+^	37.6 ± 2.18 *^,+^	35.67 ± 6.64 *^,+^	35.50 ± 9.50 *^,+^	35.33 ± 2.91 *^,+^
VLDL (mg/mL)	20.95 ± 2.54 ^+^	40.10 ± 4.21 *	16.66 ± 1.58 ^+^	6.27 ± 1.3 *^,+^	7.13 ± 1.33 *^,+^	7.10 ± 1.90 *^,+^	7.07 ± 0.58 *,^+^
AI	2.50 ± 0.15 ^+^	4.61 ± 0.80 *	2.69 ± 0.20 ^+^	2.29 ± 0.48 ^+^	3.02 ± 0.36	3.95 ± 0.35	2.67 ± 0.32 ^+^

Values are expressed as the mean ± SEM. *p* < 0.05 was considered as significant. * Statistically significant difference compared to normoglycemic controls. ^+^ Statistically significant difference compared to hyperglycemic controls. Total cholesterol (TC), triglycerides (TG), high-density lipoprotein (HDL), low-density lipoprotein (LDL), very low-density lipoprotein (VLDL), and atherogenic index (AI).

## Data Availability

Not applicable.

## Supplementary Materials

The following supporting information can be downloaded at https://www.mdpi.com/article/10.3390/molecules28155760/s1: Figure S1. Total ion chromatogram profile of RCHM; Figure S2. Total ion chromatogram profile of RCFM; Figure S3 Total ion chromatogram profile of F89s.
