# Supplementary material for: Evaluation of α-Glucosidase Inhibition and Antihyperglycemic Activity of Extracts Obtained from Leaves and Flowers of Rumex crispus L
Source: Molecules. 2023 Jul 30;28(15):5760. doi: 10.3390/molecules28155760 (PMC10420655; doi:10.3390/molecules28155760)
Supplement: Supplementary file 1 [file molecules-28-05760-s001.zip › molecules-2434426-supplementary.pdf]

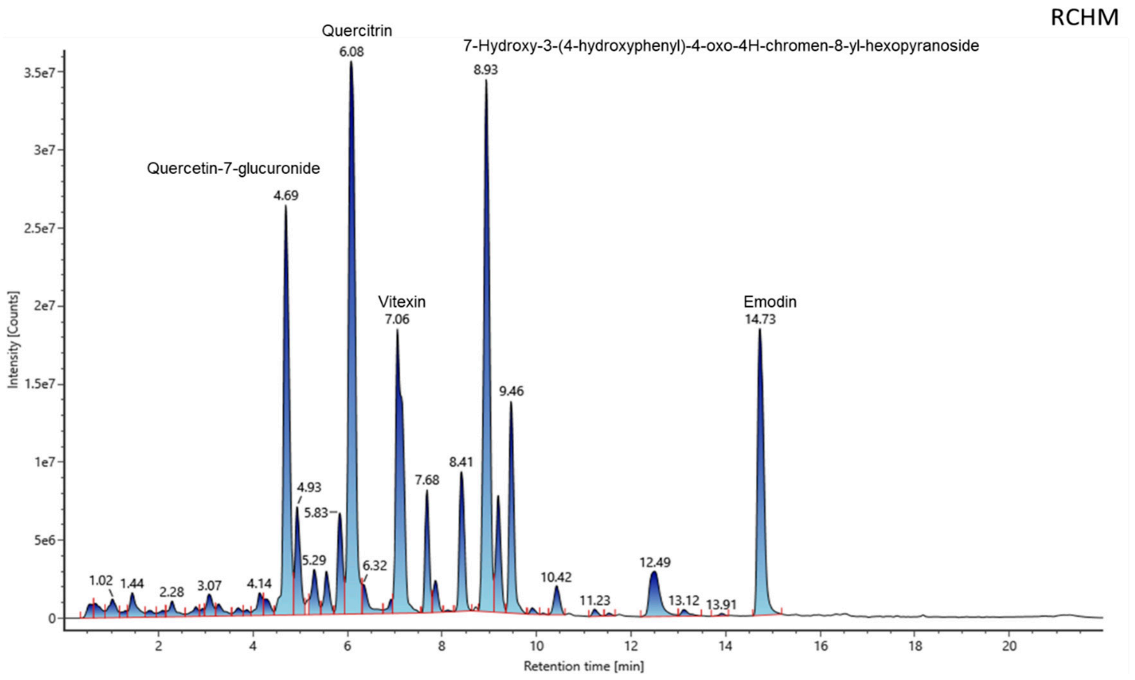

Figure S1 Total ion chromatogram profile of RCHM. For peak assignments, see Table 5

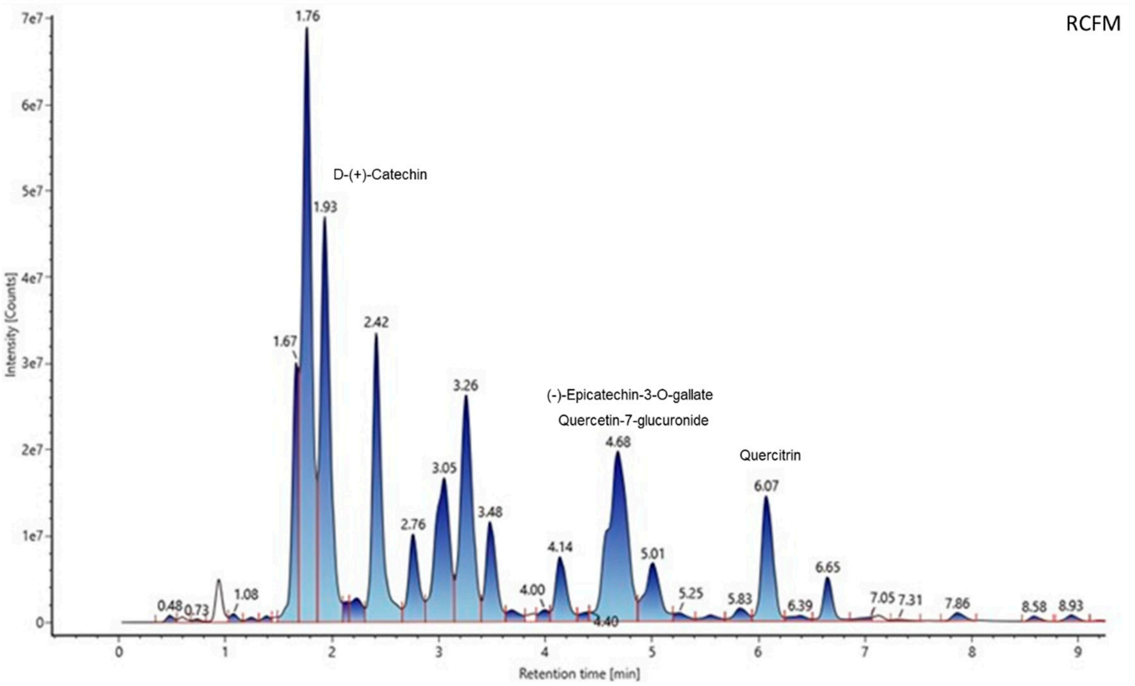

Figure S2 Total ion chromatogram profile of RCFM. For peak assignments, see Table 5

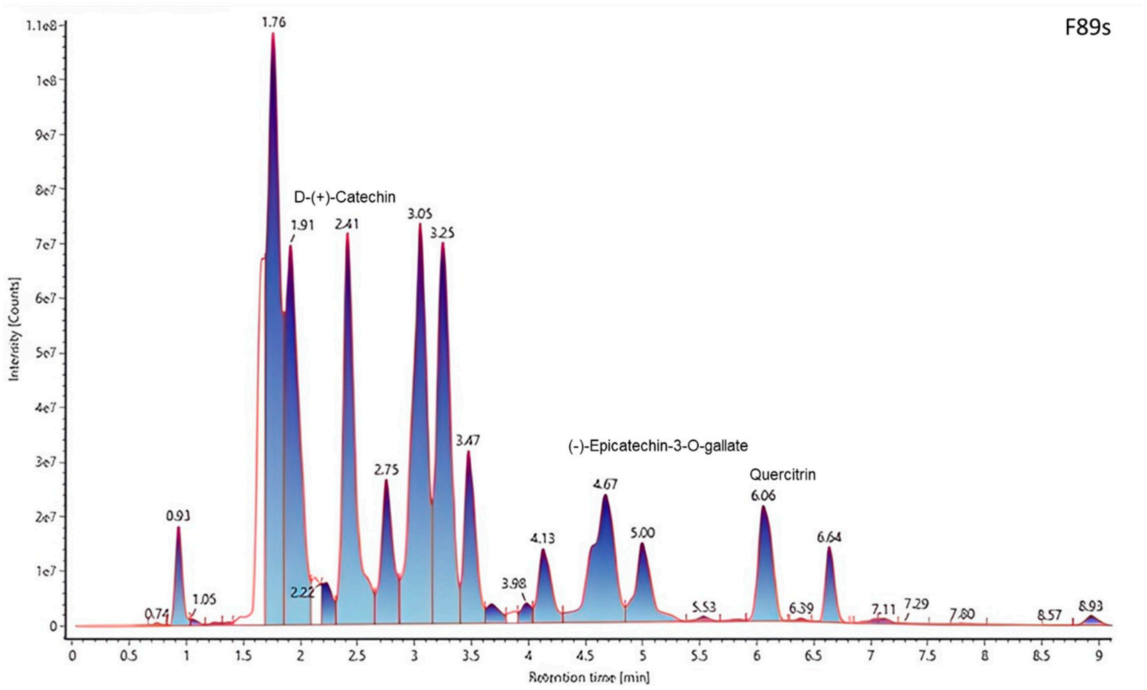

Figure S3 Total ion chromatogram profile of F89s. For peak assignments, see Table 5
